# Supplementary figures and images for: Genome-Wide Identification of Auxin-Responsive GH3 Gene Family in Saccharum and the Expression of ScGH3-1 in Stress Response
Source: Int J Mol Sci. 2022 Oct 22;23(21):12750. doi: 10.3390/ijms232112750 (PMC9654502; doi:10.3390/ijms232112750)

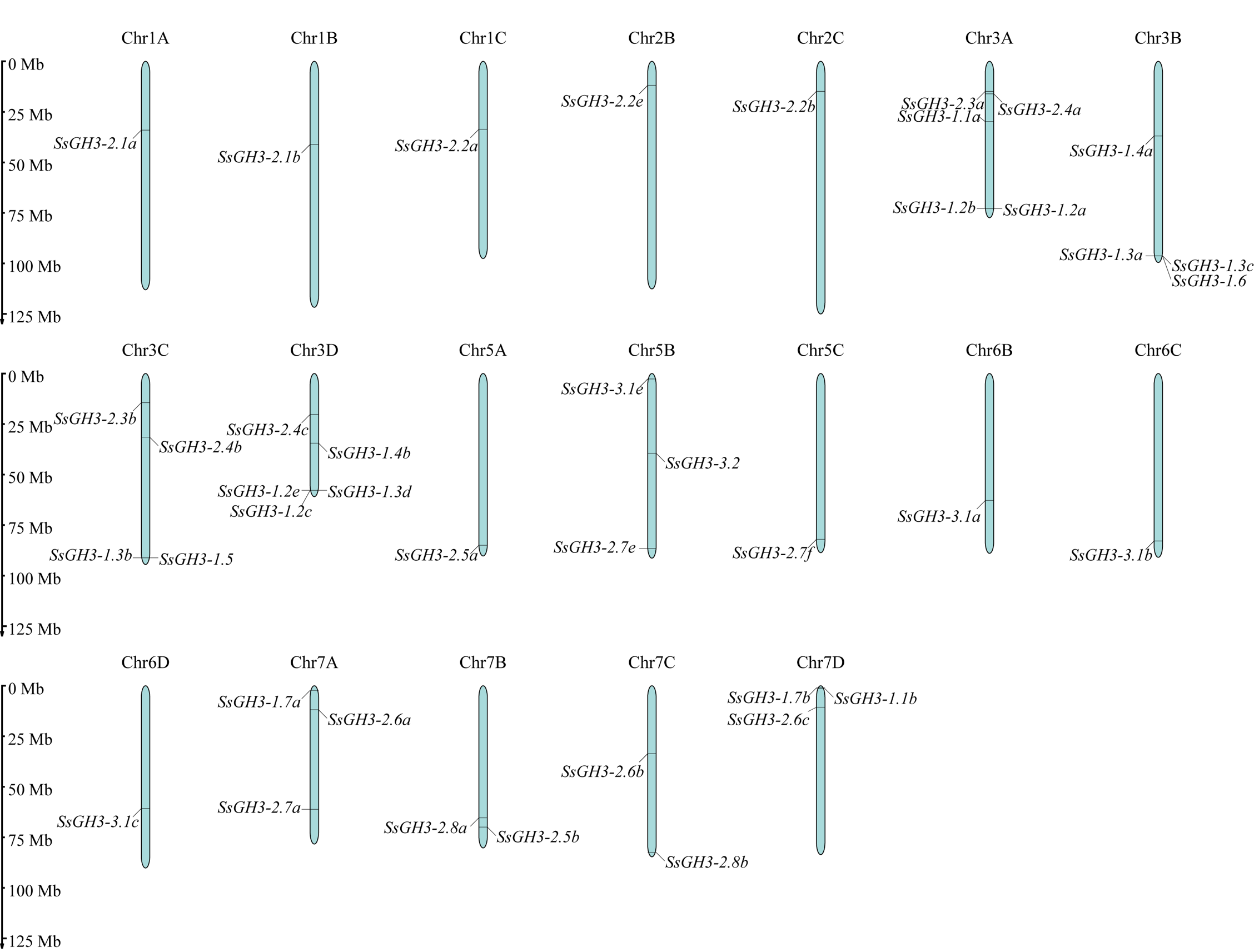

Supplement: Supplementary file 1 [file ijms-23-12750-s001.zip › Figure S1.tif]

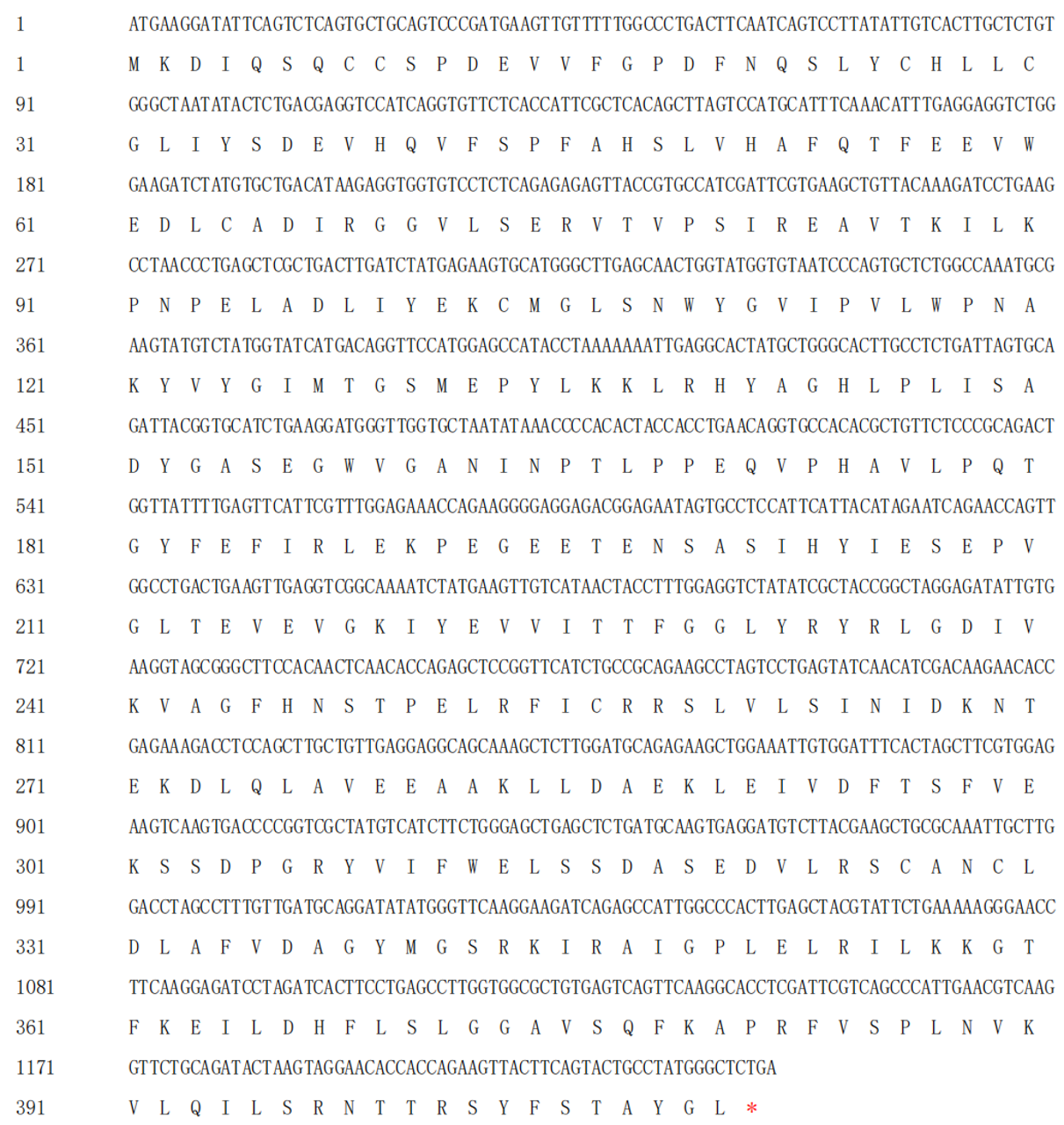

Supplement: Supplementary file 1 [file ijms-23-12750-s001.zip › Figure S2.tif]
